# Supplementary material for: Integrated Morphological, Comparative Transcriptomic, and Metabolomic Analyses Reveal Mechanisms Underlying Seasonal Patterns of Variation in Spines of the Giant Spiny Frog (Quasipaa spinosa)
Source: Int J Mol Sci. 2024 Aug 22;25(16):9128. doi: 10.3390/ijms25169128 (PMC11354522; doi:10.3390/ijms25169128)
Supplement: Supplementary file 1 [file ijms-25-09128-s001.zip › ijms-3056754-supplementary.pdf]

Table S1. Statistics of morphological indexes and spine character parameters of *Quasipaa spinosa*

Index	Min	Max	Mean	SD	CV/%
BM/g	96.25	244.00	167.52	29.86	17.81
SVL/mm	92.46	132.03	111.36	7.59	6.82
LLAH/mm	41.69	57.93	49.80	3.52	7.08
CLA/mm	51.02	82.62	66.14	6.18	9.35
HL/mm	21.14	34.4	26.80	2.54	9.48
LL/mm	117.91	156.47	142.37	7.43	5.22
TL/mm	43.62	64.77	54.76	4.57	8.35
TW/mm	16.86	24.81	20.86	1.63	7.82
FL/mm	32.39	66.54	47.35	4.92	10.39
SD/%	7.06	26.26	14.65	3.81	26.06
ABH/mm	0.74	1.61	1.09	0.19	17.70

Table S2. Pearson correlation analysis of morphological indices and spines characters of *Q. spinosa*

Trait	BM/g	SVL/mm	LLAH/mm	CLA/mm	HL/mm	LL/mm	TL/mm	TW/mm	FL/mm	SD/%	ABH/mm
M/g	1										
SVL/mm	0.823**	1									
LLAH/mm	0.591**	0.599**	1								
CLA/mm	0.647**	0.480**	0.389**	1							
HL/mm	0.581**	0.453**	0.564**	0.531**	1						
LL/mm	0.718**	0.639**	0.427**	0.475**	0.353**	1					
TL/mm	0.586**	0.555**	0.347**	0.382**	0.307**	0.557**	1				
TW/mm	0.758**	0.574**	0.521**	0.434**	0.562**	0.509**	0.351**	1			
FL/mm	0.558**	0.403**	0.455**	0.432**	0.544**	0.536**	0.299**	0.448**	1		
SD/%	-0.552**	-0.128	-0.463**	-0.626**	-0.450**	-0.252	-0.039	-0.443**	-0.348*	1	
ABH/mm	0.055	0.096	-0.219*	0.045	0.066	0.089	0.166	-0.147	-0.029	-0.343**	1

Note: ** indicates a significant correlation at the level of 0.01 (bilateral). * represents a significant correlation at the level of 0.05 (bilateral).

Table S3. Statistical table of transcriptome sequencing data

Sample	Clean Reads	GC Content	Q30 ratio	Mapped Reads (ratio)
AMC1	23,013,459	45.76 %	93.33 %	17,442,167 (75.79 %)
AMC2	23,347,527	45.89 %	93.43 %	17,462,711 (74.79 %)
AMC3	25,054,914	45.87 %	93.77 %	18,130,131 (72.36 %)
AFC1	23761464	46.33 %	94.12 %	17,796,009 (74.32 %)
AFC2	22,966,610	46.19 %	93.15 %	17,297,752 (75.32 %)
AFC3	24,956,318	46.69 %	95.53 %	18,294,266 (73.31 %)
AMA1	22,684,957	46.74 %	95.47 %	15,848,423 (71.31 %)
AMA2	22,652,514	47.55 %	94.41 %	15,750,439 (69.53 %)
AMA3	22,737,521	47.31 %	93.71 %	15,775,027 (70.09 %)
BMC1	27,571,699	46.32 %	93.30 %	20,582,715 (74.65 %)
BMC2	27,648,472	47.79 %	93.25 %	20,658,211 (75.31 %)
BMC3	27,596,055	46.06 %	90.79 %	20,678,703 (75.82 %)
BFC1	19,504,300	46.20 %	92.54 %	14,075,851 (72.17 %)
BFC2	20,617,037	46.42 %	91.73 %	14,125,250 (72.80 %)
BFC3	19,582,271	45.46 %	93.47 %	14,163,219 (72.34 %)
BMA1	29,848,320	46.15 %	93.19 %	21,871,922 (73.28 %)
BMA2	29,922,458	46.58 %	93.78 %	21,967,974 (72.94 %)
BMA3	29,857,926	46.53 %	93.62 %	21,929,598 (73.19 %)
CMC1	33,794,991	45.92 %	93.29 %	23,911,540 (70.75 %)
CMC2	33,874,145	45.47 %	93.53 %	23,925,474 (70.69 %)
CMC3	33,803,432	45.25 %	93.95 %	23,995,590 (70.93 %)
CFC1	23,176,102	45.78 %	93.15 %	16,440,516 (70.94 %)
CFC2	23,192,041	45.29 %	93.08 %	16,518,284 (70.13 %)
CFC3	23,184,659	45.83 %	93.61 %	16,445,721 (70.67 %)
CMA1	23,162,316	45.28 %	92.65 %	17,116,770 (73.90 %)
CMA2	23,158,446	45.64 %	91.83 %	17,205,912 (73.96%)

CMA3	23,169,160	45.70 %	92.51 %	17,184,778 (73.39%)

Table S4. Statistics on the number of DEGs

DEG Set	All DEGs	Up-regulated	Down-regulated
AFC_vs_AMC	1674	955	719
BFC_vs_BMC	1829	820	1009
CFC_vs_CMC	1131	773	358
AMA_vs_AMC	825	608	217
BMA_vs_BMC	769	519	250
CMA_vs_CMC	823	633	190

Table S5. KEGG enrichment pathways of DEGs of *Q. spinosa*

Group	ID	Pathway	Number of DEGs	Total number	P-value
AFC_vs_AMC	ko04512	Extracellular matrix receptor interactions	20	119	2.19E-06
	ko04260	Myocardial contraction	16	100	4.30E-05
	ko00350	Tyrosine metabolism	13	17	4.56E-05
	ko00982	Drug metabolism cytochrome P450	10	63	0.001247
	ko04060	Cytokines and their receptor interactions	19	177	0.001746
	ko04510	Cell Adhesion	22	252	0.009945
BFC_vs_BMC	ko04260	Myocardial contraction	10	100	2.67E-06
	ko04261	Adrenergic signaling	17	167	1.91E-05
	ko04020	Calcium signaling pathway	20	250	0.000117
	ko04310	Wnt signaling pathway	16	197	0.003761
	ko04145	Phagosomes	16	247	0.005004
	ko04916	Melanin synthesis	9	118	0.006975
CFC_vs_CMC	ko04060	Cytokines and their receptor interactions	25	177	4.76E-05
	ko04620	Toll-like receptor signaling pathway	14	95	0.001441
	ko01230	Amino acid biosynthesis	15	108	0.001821
	ko04512	Extracellular matrix receptor interactions	16	119	0.001836
	ko04066	HIF-1 signaling pathway	5	10	0.001987
	ko00010	Glycolysis and gluconeogenesis	12	79	0.002382

AMA_vs_AMC	ko04260	Myocardial contraction	20	100	1.43E-12
	ko04020	Calcium signaling pathway	23	250	2.35E-07
	ko04261	Cardiomyocyte adrenergic signaling	18	167	5.14E-07
	ko04512	Extracellular matrix receptor interactions	15	119	6.39E-07
	ko04510	Adhesion plaques	19	252	4.83E-05
BMA_vs_BMC	ko05414	Dilated cardiomyopathy	5	18	8.89E-05
	ko04260	Myocardial contraction	13	100	1.40E-06
	ko04261	Adrenergic signaling	16	167	5.27E-06
	ko04020	Calcium signaling pathway	16	250	0.000662
	ko04916	Melanin synthesis	8	118	0.010702
	ko04512	Extracellular matrix receptor interactions	8	119	0.011228
	ko04350	Wnt signaling pathway	6	100	0.043071
CMA_vs_CMC	ko04972	Pancreatic secretion	9	17	1.57E-10
	ko04260	Myocardial contraction	17	100	1.44E-09
	ko00010	Glycolysis and gluconeogenesis	9	79	0.000300
	ko04974	Protein digestion and absorption	5	25	0.000515
	ko04975	Fat digestion and absorption	3	10	0.002150
	ko01230	Amino acid synthesis	9	108	0.002871

Table S6. DEGs of *Q. spinosa*

Group	Gene ID	Name	Function	Log_2FC	Category
AFC_vs_AMC	c175004.graph_c0	*COL1A1*	Type I collagen	5.120	Collagen fiber tissue composition
	c185493.graph_c0	*COL1A2*	Type I collagen	3.899	Collagen fiber tissue composition
	c192649.graph_c0	*COL6A3*	Type VI collagen	2.861	cell adhesion
	c200972.graph_c0	*ITGA2*	integrins	1.176	Signal Transduction
	c189207.graph_c0	*ITGB7*	integrins	1.190	Signal Transduction
	c202023.graph_c0	*TNXB*	tenasin	1.526	Signal Transduction
	c193625.graph_c3	*KRT1*	Keratin	15.058	Keratin synthesis
	c192342.graph_c0	*TYR*	tyrosinase	7.071	melanin production
	c158711.graph_c1	*TNNT3*	Troponin	2.335	skeletal muscle synthesis
	c191109.graph_c2	*VDR*	Vitamin D3 receptor	-1.930	Inhibit cell proliferation and differentiation
BFC_vs_BMC	c186373.graph_c1	*Wnt5*	Wnt family	1.784	Signal Transduction
	c165895.graph_c0	*BMP*	bone morphogenetic protein	2.346	Cell Differentiation
	c198815.graph_c0	*Lgr6*	G protein coupled receptor	1.727	Cell Differentiation
	c200147.graph_c0	*TYR*	tyrosinase	1.524	melanin synthesis
	c182579.graph_c0	*TYRP1*	melanosome enzyme	4.019	melanin synthesis
	c177928.graph_c0	*DCT*	dopachrome isomerase	2.862	melanin synthesis
	c193625.graph_c3	*KRT1*	Keratin	15.040	Keratin synthesis
	c194666.graph_c0	*KRT17*	Keratin	2.018	Keratin synthesis
	c143894.graph_c1	*KRT6A*	Keratin	3.969	Keratin synthesis

	c172567.graph_c1	*MYH7B*	myosin	7.049	material transport
	c178547.graph_c1	*MYL10*	myosin	6.747	material transport
	c191135.graph_c0	*DNH3*	dynein	1.174	material transport
	c199992.graph_c2	*PAK3*	phosphorylated protein kinase	1.013	cell movement
	c192934.graph_c0	*CLDN8*	connexin	9.623	Related protein synthesis
	c172679.graph_c0	*TECTA*	coat protein	8.026	Related protein synthesis
	c173496.graph_c0	*SFRP5l*	secreted Frizzled protein	7.536	Related protein synthesis
	c50612.graph_c0	*TLR1*	Toll-like receptor	2.599	immunity
	c183205.graph_c0	*CRABP1*	retinoic acid binding protein	-4.641	apoptosis
	c153068.graph_c0	*ALDOA*	Aldolase A	-3.801	Cell Proliferation
CFC_vs_ CMC	c153068.graph_c1	*ENO1*	multifunctional protease	-5.184	Cell division
	c193736.graph_c0	*PRPS2*	Phosphoribosylpy rophosphate synthase II	-2.328	apoptosis
	c87915.graph_c0	*GAPDH*	Olealdehyde-3-ph osphate dehydrogenase	-4.392	apoptosis
	c162826.graph_c0	*TNN*	Tenascin N	8.078	extracellular
	c175004.graph_c0	*COL1A1*	Collagen	3.166	matrix
AMA_vs_ AMC	c183505.graph_c0	*THBS4*	Thrombospond in 4	3.759	protein
	c185493.graph_c0	*COL1A2*	Collagen	2.561	extracellular
	c192649.graph_c0	*COL6A3*	Collagen	2.267	matrix

	c197928.graph_c0	*TNC*	tenascin C	2.519	structure
	c200292.graph_c0	*THBS2*	Thrombospond in 2	1.587	cell
	c170031.graph_c0	*MYPLF*	myosin light chain	7.735	adhesion
	c177928.graph_c0	*DCT*	dopachrome isomerase	1.808	extracellular
	c180793.graph_c0	*TYR*	tyrosinase	1.362	matrix
	c182579.graph_c0	*TYRP1*	melanosome enzyme	4.849	structure
	c193625.graph_c3	*KRT*	Keratin	7.294	extracellular
	c185955.graph_c0	*KRT12*	Keratin	6.468	matrix
	c143894.graph_c2	*KRT6A*	Keratin	3.633	structure
BMA_vs_ BMC	c198796.graph_c0	*KRT5*	Keratin	2.890	extracellular
	c162826.graph_c0	*TNN*	Tenascin N	4.447	matrix
	c183505.graph_c0	*THBS4*	Thrombospond in 4	3.097	protein
	c197928.graph_c0	*TNC*	tenascin C	1.528	collagen
	c153170.graph_c0	*SFRP1*	Secreted Frizzled-relate d protein	1.467	fiber
	c155621.graph_c0	*DKK1*	Dickkopf	3.326	production
	c161165.graph_c0	*FOSL1*	FOS-like antigen 1	1.245	cell

	c186343.graph_c3	*PLCB2*	Phospholipase C-β2	1.113	morphogenesis
	c186373.graph_c1	*Wnt5a*	Wnt family 5A	2.639	melanin
	c189154.graph_c0	*WISP1*	Wnt1 induced signaling pathway protein 1	1.860	synthesis
	c189853.graph_c0	*CAMKII*	calmodulin kinase	4.374	melanin
	c153068.graph_c1	*ENO1*	multifunctional protease	-4.762	synthesis
	c161619.graph_c0	*PKM2*	pyruvate kinase	-3.928	melanin
	c172589.graph_c0	*FBP2*	Fructose-1,6 bisphosphatase -2	7.903	synthesis
CMA_vs_ CMC	c177628.graph_c0	*ALDOA*	fructose bisphosphate aldolase A	2.941	Keratin
	c183063.graph_c0	*ENO3*	multifunctional protease	5.058	synthesis
	c189108.graph_c0	*PFKM*	phosphofructokinase muscle type	4.088	Keratin
	c194236.graph_c1	*PGAM2*	glycerophosphomutase	3.978	synthesis
	c182598.graph_c0	*KRT10*	Keratin	-1.099	Keratin

Table S7. Skin metabolites of *Q. spinosa* in different comparison groups

Comparable group	Differential metabolites	VIP	P-value	KEGG ID	pathwat
	Creatine	2.00	0.014	C00300	map00260
	Phthalic acid	1.99	0.006	C01606	map00624
	Alanine	1.84	0.021	C00099	map00240
BMC_vs_BMA	Maleamate	1.76	0.035	C01596	map00760
	Threo-beta-hyrdoxyaspartate	1.66	0.041	\	\
	2,4-diaminobutyric acid	1.64	0.047	K06718	map00260
	Glucose-1-phosphate	1.58	0.047	K20866	map00010
	Alpha-Tocopherol	2.22	0.000	C02477	map00130
	L-Malic acid	2.08	0.002	C00149	map00020
	D-glycerol 1-phosphate	2.07	0.004	C00093	map00561
	3-Hexenedioic acid	1.95	0.005	\	\
	Citric acid	1.91	0.015	C00158	map00020
	Xylose	1.87	0.010	C01394	\
BMC_vs_BFC	Thymidine	1.77	0.025	C00214	map00240
	Beta-Alanine	1.75	0.028	C00099	map00240
	Threo-beta-hyrdoxyaspartate	1.70	0.031	\	\
	Dihydroxyacetone	1.69	0.018	C00184	map00561
	Sorbitol	1.68	0.024	C00794	map00051
	Glucose-1-phosphate	1.66	0.040	K20866	map00010
	Phosphorylethanolamine	1.65	0.027	C00346	map00563

	Hexadecane	1.64	0.037	\	\
	Beta-Mannosylglycerate 2	1.60	0.043	\	\
	Alpha-ketoglutaric acid	1.59	0.039	C00026	map00020
	Behenic acid	1.58	0.033	C08281	map00073
	Carbobenzyloxy-L-leucine degr1	1.51	0.041	\	\
